# Red Imported Fire Ant (*Solenopsis invicta*) Chemosensory Proteins Are Expressed in Tissue, Developmental, and Caste-Specific Patterns

**DOI:** 10.3389/fphys.2020.585883

**Published:** 2020-10-23

**Authors:** Arun Wanchoo, Wei Zhang, Almudena Ortiz-Urquiza, John Boswell, Yuxian Xia, Nemat O. Keyhani

**Affiliations:** ^1^Department of Microbiology and Cell Science, Institute of Food and Agricultural Sciences, University of Florida, Gainesville, FL, United States; ^2^Genetic Engineering Research Center, School of Life Sciences, Chongqing University, Chongqing, China; ^3^Department of Biosciences, College of Science, Swansea University, Swansea, United Kingdom

**Keywords:** social insects, chemical communication, ligand binding proteins, chemosensory proteins, caste, tissue, developmental expression

## Abstract

The red imported fire ant, *Solenopsis invicta*, is a eusocial invasive insect that has spread worldwide. Chemosensory proteins (CSPs) are ligand-binding proteins that participate in a diverse range of physiological processes that include olfaction and chemical transport. Here, we performed a systematic survey of the expression of the 21 gene *S. invicta CSP* family that includes at least two groups of apparent *S. invicta*-specific gene expansions. These data revealed caste, tissue, and developmental stage-specific differential expression of the *SiCSPs*. In general, moderate to high *SiCSP* expression was seen in worker antennae and abdomen tissues with lower expression in head/thorax regions. Male and female alates showed high antennal expression of fewer *SiCSPs*, with the female alate thorax showing comparatively high *SiCSP* expression. *SiCSP* expression was lower in male alates tissues compared to workers and female alates, albeit with some highly expressed *SiCSPs*. *SiCSP* expression was low during development including in eggs, larvae (early and late instars), and pupae. Global analyses revealed examples of conserved, divergent, and convergent *SiCSP* expression patterns linked to phylogenetic relationships. The developmental and caste-specific variation seen in *SiCSP* expression patterns suggests specific functional diversification of CSPs that may translate into differential chemical recognition and communication among individuals and/or reflect other cellular roles of CSPs. Our results support a model for CSPs acting as general ligand carriers involved in a wide range of physiological processes beyond olfaction. As compared to the expression patterns of the *S. invicta* odorant binding proteins (OBPs), an inverse correlation between *SiOBP* and *SiCSP* expression was seen, suggesting potential complementary and/or compensatory functions between these two classes of ligand carriers.

## Introduction

Ants are the most numerous of the social insects and one of the most successful animals on earth (Holldobler and Wilson, 1990). The red imported fire ant, *Solenopsis invicta* (Buren) was originally endemic to South America (Northern Argentina, Southern Brazil, and parts of Paraguay). Sometime between 1933 and 1945, this ant likely found its way aboard ships carrying exotic fruits, lumber, and other goods from South America apparently establishing somewhere around Mobile, Alabama in the United States, from where it has spread to many other parts of the world (Tschinkel, 2006; Shoemaker et al., 2011). In this respect, *S. invicta* has demonstrated a remarkable plasticity in environmental adaptations making it one of the most successful invasive animal species on our planet. It is an important pest throughout the southern United States and has recently invaded and established itself in California, South East Asia, and even parts of Europe. *S. invicta* form complex societies that are territorial, and whose members display strong nest-mate recognition, and elaborate task specialization (Tschinkel, 2006). Their colonies are considered to form a multi-tiered “caste” system consisting of non-reproductive workers, that display task-differentiation, and reproductive winged males and females. Male and female reproductive forms are produced seasonally as the colony matures, a process that can take up to 5 years. Depending upon their genetic background *S. invicta* colonies can have one (monogyny) or multiple queens (polygyny) (Ross and Fletcher, 1985; Gotzek and Ross, 2008). These two differing colony organizations are genetically distinct and have consequent important differences in the biology and responses of *S. invicta*. Originally linked to a single gene, termed *Gp-9* (renamed OBP3), mono- versus polygyne colony organization appears to be linked to a larger 13 Mb non-recombining “social” chromosomal fragment (∼55% of the chromosome) containing at least 616 open reading frames (Wurm et al., 2011; Wang et al., 2013).

Chemosensory proteins (CSPs) are a protein family defined by amino acid homology and sequence motifs that include four cysteine residues (two disulfide bridges) with conserved spacing and a set of α-helices that form a hydrophobic binding cavity as part of a compact, small molecular weight (10–15 kDa) structure (Lartigue et al., 2002; Zhou et al., 2006; Pelosi et al., 2014). CSPs were originally identified as a protein accumulating during leg regeneration in nymphal stages of the cockroach *Periplaneta americana* (Nomura et al., 1992), other CSPs were subsequently found enriched in sensory organs including the antennae, and hence presumed to function within the context of chemoreception or olfaction by acting as odorant (ligand) carrier proteins (Angeli et al., 1999; Picimbon et al., 2000; Ban et al., 2003). CSPs in the alfalfa plant bug (*Adelphocoris lineolatus*) have been implicated in mediating host recognition (Gu et al., 2012), and CSP/Takeout genes have been shown to be involved in olfactory-based behaviors including repulsion and attraction in the migratory locust (*Locusta migratoria*) (Guo et al., 2011). However, it has also been recognized that many CSPs are expressed in a variety of other tissues, e.g., in the pheromone glands of the cabbage moth, *Mamestra brassicae* and the silk moth, *Bombyx mori*, and in male and female reproductive organs, e.g., ejaculatory bulb of *Drosophila melanogaster* and female organs of *L. migratoria manilensis* (Zhou et al., 2013), where CSPs potentially function in pheromone detection and release (Jacquin-Joly et al., 2001; Dani et al., 2011). More recently, various CSPs have been implicated in a wide range of physiological processes beyond olfaction and/or chemical communication. RNAi knockdown of the honeybee (*Apis mellifera*) CSP5 indicated a role for this protein in embryonic integument development (Maleszka et al., 2007) and CSP3 (designed ASP3c), also from the honeybee, has been shown to act as a brood pheromone carrier protein (Briand et al., 2002). CSPs, therefore, should be considered as general ligand carrier proteins, some of which may function within the scope of chemical perception mediating binding of volatile and/or hydrophobic odorants, whereas others may ferry hormones and/or other chemical compounds participating in organismal homeostasis and/or developmental process. Participation in pheromone/semiochemical sequestration and/or release would still link CSPs to chemical communication, albeit in the broad sense, namely outside of sensillar mediated olfaction.

Chemosensory proteins are considered to have at least some parallels in function to another class of small molecular weight (∼130–150 amino acids), soluble proteins known as odorant binding proteins (OBPs). OBPs, like CSPs, are thought to mediate signal transduction in insects by shuttling hydrophobic compounds (e.g., odorants) to odorant receptors expressed on the dendritic membrane of the olfactory neurons. Insects OBPs do not share homology to the vertebrate OBPs and are sometimes annotated as pheromone binding proteins (PBPs) or general odorant binding proteins (GOBPs) (Zhang et al., 2016). Expression profiling of the *S. invicta* 17-gene member OBP family revealed both antennal and non-antennal expression specific *SiOBPs*, as well as caste and tissue specificity in the expression of these proteins (Zhang et al., 2016). Intriguingly, the *S. invicta* “social” chromosomal fragment contains multiple members of both the CSP and OBP gene families amongst other genes.

Antenna-specific CSPs, enriched in specialized chemosensory sensilla, have been identified in the Argentine ant (*Linepithema humile*) and the Japanese carpenter ant (*Camponotus japonicus*), with CjCSP1 mediating cuticular hydrocarbon recognition and transport, a critical process for nestmate/non-nestmate discrimination in *C. japonicus* (Ishida et al., 2002; Ozaki et al., 2005). Comparative transcriptomics in a variety of ant species also indicated expression of CSPs in non-antennal tissues, further supporting their functioning beyond olfaction (McKenzie et al., 2014). Initially, fourteen CSP sequences were identified in *S. invicta* expressed sequence tag (EST) libraries, with one, designated SiCSP1(SiCSP19 in our study), as well as an apolipophorin-like protein, found to be highly expressed in the worker antennae (Guntur et al., 2004; Gonzalez et al., 2009; Xu et al., 2009). Subsequent genome mining and phylogenetic analyses revealed intriguing CSP gene expansions in the available ant genomes with the apparent evolution of ant-specific CSP clades (Kulmuni et al., 2013). Within these analyses 21 CSPs in total were identified in the *S. invicta* genome, representing one of the largest CSP sets annotated in eusocial Hymenoptera to date. Most *Drosophila* spp., by contrast, have only four CSP genes (Vieira and Rozas, 2011).

Here, the expression pattern of the 21 CSP genes identified in *S. invicta* was examined by quantitative reverse transcriptase polymerase chain reaction (qRT-PCR). *SiCSP* gene expression patterns were examined as a function of caste, tissue distribution, and developmental stage. Developmental specificity of *SiCSP* was examined in eggs, early instar larvae, late instar larvae, and pupae. For adult ant (workers, male and female alates) samples were further subdivided into specific anatomical tissues that included the antenna, head, thorax, and abdomen. High to robust expression of most *SiCSPs* was found in the antennae of workers and male alates, and to a lesser extent, in female alate antennae. *SiCSP* expression was significantly lower in the developmental stages as compared to the adults, although robust expression of specific *SiCSPs* were found in early-instar larvae and pupae. These data revealed dynamic and differential *SiCSP* expression patterns among developmental stages and adult tissues with little correlation to their phylogenetic relatedness, although sub-groups of phylogenetically related *SiCSPs* showed similar patterns of expression. Both divergence between more closely related SiCSPs and convergence between more remotely related SiCSPs was observed. This study provides a basis for a further systematic characterization of the functions of the different CSPs in *S. invicta*.

## Materials and Methods

### Insects and Experimental Samples

*Solenopsis invicta* laboratory colonies were collected from the field (Gainesville, FL, United States) and maintained as described in Fan et al. (2012). The field colony was assessed to be polygyne with multiple queens evident in the founding colony. In addition, sequencing of the full-length cDNA of *SiOBP3/Gp-9* gene revealed the presence of both *Gp-9B* and *Gp-9b* alleles. Laboratory colonies were maintained at room temperature with ∼70% relative humidity and a 16:8 dark:light photoperiod. Ants were fed with 300 mM sucrose solutions randomly dispersed throughout the trays and supplemented with freeze dried *Galleria mellonella* larvae. Dissections into four sections; antenna, head (without antennae), thorax and abdomen were performed using separately processed (∼200–500 ants) workers, male and female alates that were immersed in RNALater (Invitrogen, Thermo Fisher Scientific, Waltham, MA, United States) and dissected under a stereomicroscope. Adult stages were not sampled as same-age cohorts. Eggs, collected from mated queens within 24 h, as well as small larvae, large larvae and pupa (approximately 200–500 each) were immediately suspended in RNALater and stored at −80°C until RNA extraction. Larvae were distinguished by their size and reflected a mixture of minor and major workers that could include male and female alates. Early instars (1st and 2nd) were selected based on size (≤1 mm) and color, as these often displayed some melanization, whereas late instars (3rd and 4th) were larger (1.1–4.5 mm) and clear to whitish.

### RNA Preparation and cDNA Library Construction

Samples (∼100 mg) were ground in liquid nitrogen using a mortar and pestle, after which Trizol reagent (1 ml) was added, and total RNA extracted following the manufacturers’ protocols (Invitrogen, Carlsbad, CA, United States). Genomic DNA in samples were digested using TURBODNase (Invitrogen, Carlsbad, CA, United States). Total RNA quality and quantity were analyzed by agarose gel electrophoresis and via NanoDrop 2000 spectrophotometric analyses. Quantification of RNA concentrations in samples was performed using a Qubit H 2.0 fluorometer (Invitrogen, Carlsbad, CA, United States) and cDNA libraries were constructed using 2 mg total RNA using the High-Capacity cDNA Reverse Transcription Kit (Applied Biosystems, Foster City, CA, United States). At least three biological replicates were prepared for each sample.

### Absolute Qualification of cDNAs

The amino acid sequences of the *S. invicta* chemosensory proteins (SiCSPs) were downloaded from NCBI (Supplementary Table S1). Absolute qualification of the *SiCSPs* were performed as previous described (Whelan et al., 2003). Briefly, target-specific qRT-PCR primers were designed using the Beacon designer 8.13 software program (Palo Alto, CA, United States) and synthesized commercially (Invitrogen, Carlsbad, CA, United States). The list of primers used is given in Supplementary Table S2. Primers were used in PCR reactions to amplify *SiCSP* target sequences using an *S. invicta* cDNA library constructed as described above as the template. After purification, PCR fragments were cloned into the pGEMT vector (Promega Corp., Madison, WI, United States). Positive clones were isolated, and the integrity of the inserts verified by sequencing (Eton Biosciences, San Diego, CA, United States). Concentrations of the pGEMT plasmids for each *SiCSP* were quantified using Qubit dsDNA BR Assay Kit (Invitrogen, Carlsbad, CA, United States) for use in constructing absolute quantification standard curves. RT-PCR primer sets for each *SiCSP* were validated for production of the correct amplicon size, optimal ratios of primers, *T*_m_, and efficiency (Supplementary Table S3). The amplification efficiency (E) was calculated using the slope of a linear regression determined by the Ct values (*Y*-axis) and the log_10_ concentration of the cDNA (*X*-axis). Slopes were used to calculated Efficiencies (*E*) using the formula: *E* = 10^1/slope^−1. Plasmid constructs and optimized PCR conditions were used to acquire data for construction of absolute expression standard curves, using serial dilution of the plasmid templates (10^–5^ to 10^–9^). The number of transcript copies was calculated using the molecular weight of plasmids and their empirically determined concentrations using the formula: copy number = (6.02 × 10^23^) × (amount ng × 10^–9^)/(DNA length × 660).

Individual standard curves and empirically determined C_t_ values (as derived from the qRT-PCR experiments) were used to calculate *SiCSP* absolute transcript expression values. qRT-PCR reactions were performed using 2XSYBR Green qPCR Master Mix (Biotools, Houston, TX, United States). In most cases, *S. invicta* cDNA libraries were diluted 40-fold in sterile RNAase-free H_2_O. Typically, reactions included: 2XMaster Mix (7.5 ml), 5 μL template (12.5 ng of cDNA) and 200 nM of each gene specific primer in a total volume of 15 ml. qRT-PCR reactions were performed for at least three RNA preparations (i.e., three independent biological samples) from each tissue sample. PCR reactions were performed using the Eco Real-Time qPCR System (Illumina, San Diego, CA, United States) with a thermo-profile of one cycle of 95°C 5 min, 95°C 2 min, then 45 cycles of 95°C 15 s, and 60/59°C 45 s, followed by a melting curve analysis from 55 to 95°C. Absolute quantification of the *S. invicta* elongation factor-α (*EF1*α) and glyceraldehyde 6-phosphate dehydrogenase (*GAPDH*) genes of *S. invicta* were used in normalization analyses, and the geometric mean of the absolute number of transcripts of *EF1*α and *GAPDH* in every examined tissue was used to obtain the values of normalized relative mRNA transcript abundance.

### Data Analysis

Analysis of variance and multivariate analysis of the variance (ANOVA and MANCOVA) were performed to compare *SiCSP* expression levels among adult tissues and across developmental stages. The data, normalized relative mRNA transcript abundance, were transformed logarithmically (Log_10_) in order to correct normality and unequal variances. Means were compared with Tukey’s HSD (honestly significant differences) test. Statistical analyses were performed using the IBM SPSS Statistics, version 26 (Armonk, NY, United States). Expression profiles for eggs, larvae and pupae were assessed using one-way ANOVA with *post hoc* comparisons using Tukey’s HSD test within and across developmental stages. CSP expression in adults were analyzed via MANCOVA (two variables: caste and tissue) and Tukey’s HSD test was performed for each examined tissue among castes. Co-expression patterns of CSPs genes among developmental stages and among adult tissues were inferred by unsupervised hierarchical clustering of the log-transformed normalized expression data. Clustering analyses were implemented by rows (i.e., *CSP*) and columns (i.e., tissue/developmental stage) using the web-based tool Morpheus^1^. The similarity between the objects in the matrix was assessed with the one minus Pearson’s correlation metric and the complete linkage approach.

### Phylogenetic and Motif Analyses

The amino acid sequences of a total of 92 CSPs from *S. invicta*, *Acromyrmex echinatior*, *C. floridanus*, *C. japonicus*, *Harpegnathos saltator*, *D. melanogaster*, *D. grimshawi*, *Apis mellifera*, and *Polistes canadensis* were used for phylogenetic tree construction and motif analyses (Supplementary Table S4). Putative N-terminal signal peptides were identified using Signal P^2^ (Supplementary Table S1). Exon-intron splice positions were identified using the online Gene Structure Display Server (GSDS^3^). Amino acid motifs were identified using MEME (version 4.11.2^4^). The amino acid multiple sequence alignment (MSA) was generated with PRANK (Löytynoja and Goldman, 2005, 2010). The best fitting model of amino acid substitution was estimated with MEGA 6.0 (Tamura et al., 2013). MEGA optimizes the tree topology search starting with a Neighbor Joining tree and uses the likelihood function and three model criteria BIC (Bayesian Information Criterion), AIC (Akaike information criterion) and LnL (log likelihood) to find the best fitting model of amino acid substitution, which was: LG + G (G = Gamma shape parameters). The phylogenetic tree was built using RaxML at the CIPRES Science Gateway (Miller et al., 2010; Stamatakis, 2014) and the LG amino acid substitution model. G was estimated, branch lengths optimized, and branch support calculated by bootstrapping. RaxML was allowed to execute 1000 rapid bootstrap inferences and halt bootstrapping automatically after a thorough maximum likelihood search (893 bootstraps). The software MEGA 6.0 was used to draw the tree.

## Results

### The Chemosensory Protein Repertoire of *Solenopsis invicta*

**FIGURE 1:**
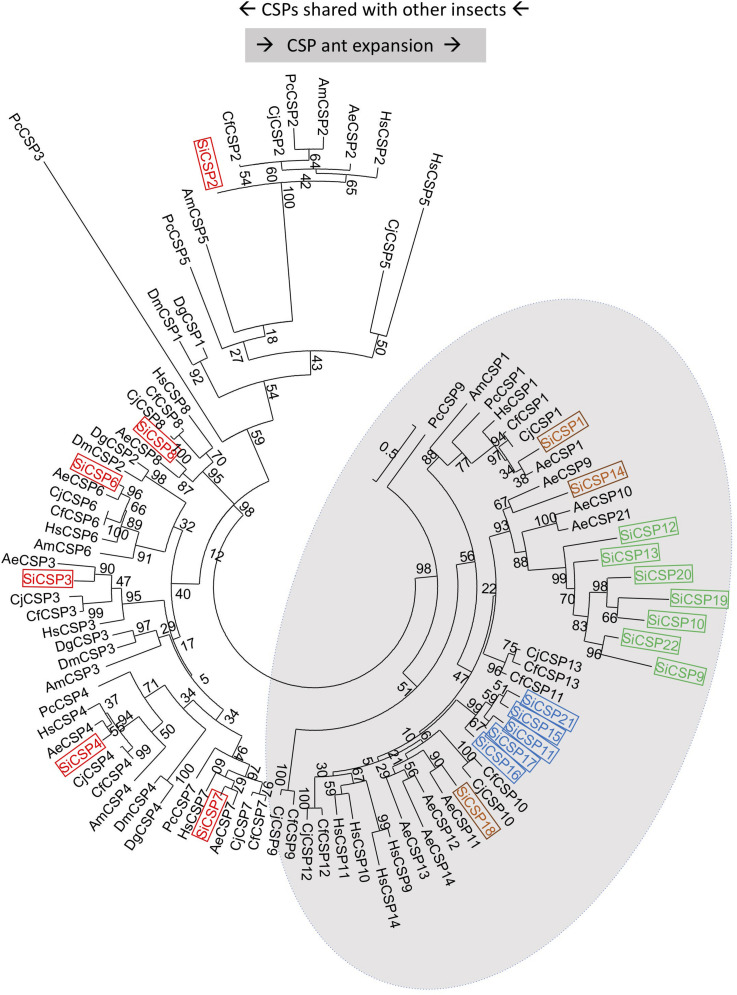
Phylogenetic analysis of *S. invicta* chemosensory proteins (SiCSPs). Limited maximum likelihood phylogeny of *S. invicta* CSPs compared to the CSP repertoires found in the ant species, *A. echinatior* (AeCSP), *C. floridanus* (CfCSP), *C. japonicus* (CjCSP), and *H. saltator* (HsCSP), the European honeybee *Apis mellifera*, the primitive eusocial wasp *Polistes canadensis* and in the fruit flies *D. melanogaster* (DmCSP) and *D. grimshawi* (DgCSP) (accession numbers given in Supplementary Table S4). Numbers at nodes indicate bootstrap values. The tree is midpoint-rooted in the absence of a suitable out-group.

A set of 21 *CSP* genes has been previously identified in the genome of *S. invicta* (Supplementary Table S1). A limited protein sequence based phylogenetic tree illustrates the division of these CSPs into two discrete major branches, one containing those showing orthology to sequences found in other insects, i.e., CSPs 8, 7, 2, 6, 3, and 4 (boxed in red), which we henceforth term “general CSPs,” and those found essentially only in ants albeit with some exceptions: “ant CSP expansion” (Figure 1). Within the ant expansion grouping, two *S. invicta*-specific gene expansions were apparent; namely one consisting of *SiCSPs 19* [identified as a major antennal SiCSP (Gonzalez et al., 2009)], *9*, *10*, *12*, *13*, *20* and *22* (boxed in green in Figure 1) and another including *SiCSPs 11*, *15*, *16*, *17* and *21* (boxed in blue). Three additional *SiCSPs*: *1*, *14* and *18* (boxed in brown) were also found distributed within the ant expansion group forming separate sub-clades. Of note, SiCSP1 showed high similarity (83.7%) to the cuticular hydrocarbon recognition CSP identified in the Japanese carpenter ant (*C. japonicus*), labeled as CjCSP1 in Figure 1 (Ozaki et al., 2005).

**FIGURE 2:**
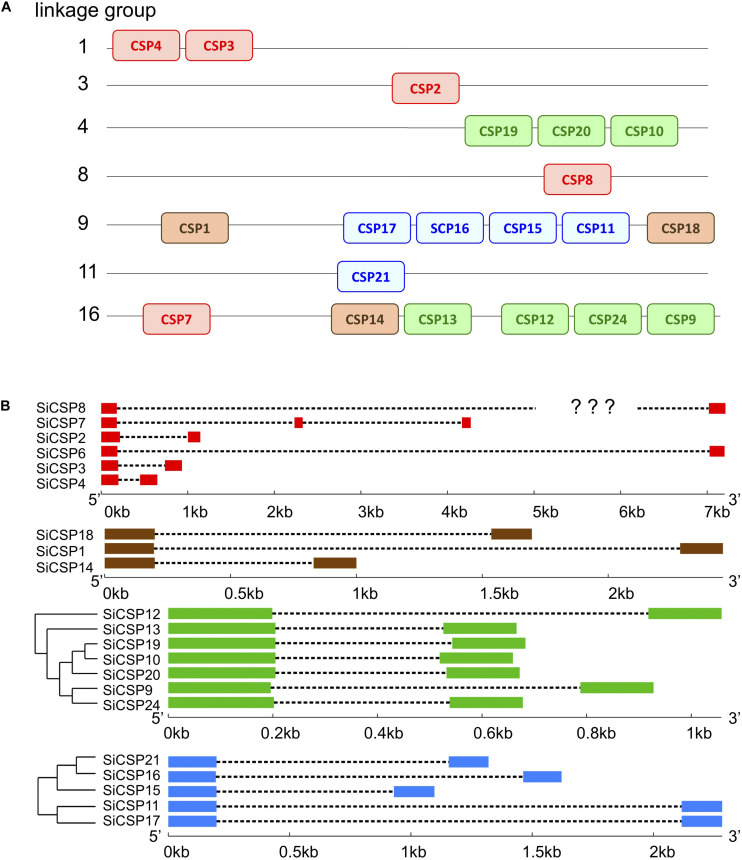
**(A)** Mapping of *SiCSP* nucleotide sequences to 16 linkage groups corresponding to the 16 *S. invicta* chromosomes (Wang et al., 2013). *SiCSP* positions are not drawn to scale but indicate approximate locations on the linkage groups. *SiCSP* exon boxes are color coded to match their phylogenetic placement as in Figure 1. **(B)** Intron–exon structures of *SiCSPs*.

Mapping of the *SiCSP* sequences to the *S. invicta* genome revealed their clustering into seven of the sixteen linkage groups reported by Wang et al. (2013) (Figure 2A, CSPs are color coded as in Figure 1). Of the general *SiCSPs*, *SiCSPs 3* and *4* (on same linkage group), *SiCSP2*, *SiCSP8*, and *SiCSP7* were located on linkage groups (Lg-) 1, 3, 8, and 16 respectively. *SiCSP7* was found on the same linkage group (Lg16) as the member of the ant expansion *SiCSP14* and four representative of one of the fire ant specific CSP gene expansion (boxed in green), namely *SiCSPs 13*, *12*, *22* and *9*. The remaining members of the latter fire ant specific expansion, i.e., *SiCSPs19*, *10* and *20*, were localized to linkage group Lg4. Four (out of five) members of the second fire ant specific CSP gene expansion (boxed in blue), namely, *SiCSPs 17*, *16*, *15*, and *11* were clustered on linkage group Lg9, along with the two remaining members of the ant CSP expansion, *CSPs 1* and *18*. The remaining member of the (blue boxed) fire ant specific CSP gene expansion, *CSP21*, was localized to linkage group Lg11. *SiCSP6* could not be assigned to any of the 16 linkage groups due to incomplete assembly.

The intron/exon structure of the *SiCSPs*, was determined using the online Gene Structure Display Server (GSDS, see text footnote 3), and correlated with *SiCSP* phylogenetic relatedness (Figure 2B). Members of the general *SiCSPs* (*2*, *3*, *4*, *6*, *7*, and *8*) showed significant variation in intron/exon structure (the intron/exon structure of *SiCSP8* could not be definitively assigned due to ambiguities in the published sequence). Aside from *SiCSP7*, which contained two introns, all other *SiCSPs* contained a single intron. The overall genomic sequences of the general *SiCSPs* ranged from ∼0.75 to >7 kb (the open reading frame sequence for each *SiCSP* is ∼300–330 bp coding for proteins ∼100–110 amino acids in length). *SiCSPs 1*, *14*, and *18* (distributed within the ant CSP expansion group) also varied in intron/exon structure. However, the overall genomic context ranged from 1 to 2.5 kb. The fire ant specific clade consisting of *SiCSPs 9, 10, 12*, *13*, *19*, *20*, and *22* were very similar in intron/exon structure, ranging in size from only ∼0.7 to 1.2 kb. Within the subgroup of *SiCSPs 10*, *19*, and *20*, which clustered together in the phylogenetic tree and were also found tandemly arrayed on linkage group Lg4, little variation in intron/exon structure was seen. Similarly, the variation of intron/exon structure seen for the second fire ant CSP gene expansion (*SiCSPs 11*, *15*, *16*, *17*, and *21*) was also low, ranging from ∼1.1 to 2.5 kb, with *SiCSP11* and *17* nearly identical.

### Chemosensory Protein Expression in *S. invicta* Adults and Developmental Stages

Oligonucleotide primers were validated for quantitative RT-PCR as described in the “Materials and Methods” section. Primer efficiency and amplification of single bands corresponding to the predicted size of each amplicon were verified (Supplementary Tables S2, S3). All amplicons were cloned and used to construct standard curves for absolute quantification of each respective transcript number in the cDNA samples. In addition to primers designed to the 21 *SiCSPs*, primers were also designed to two different “housekeeping” genes for use as references. These included primers targeting transcripts for glyceraldehyde-6-phosphate dehydrogenase (*GAPDH*) and elongation factors 1a (*EF1*α). Multivariate analysis of the variance of the normalized gene expression data showed that the examined tissues and castes, as well as the interaction between both independent variables, had a significant effect on the expression of *S. invicta CSPs* (*P* < 0.001).

**FIGURE 3:**
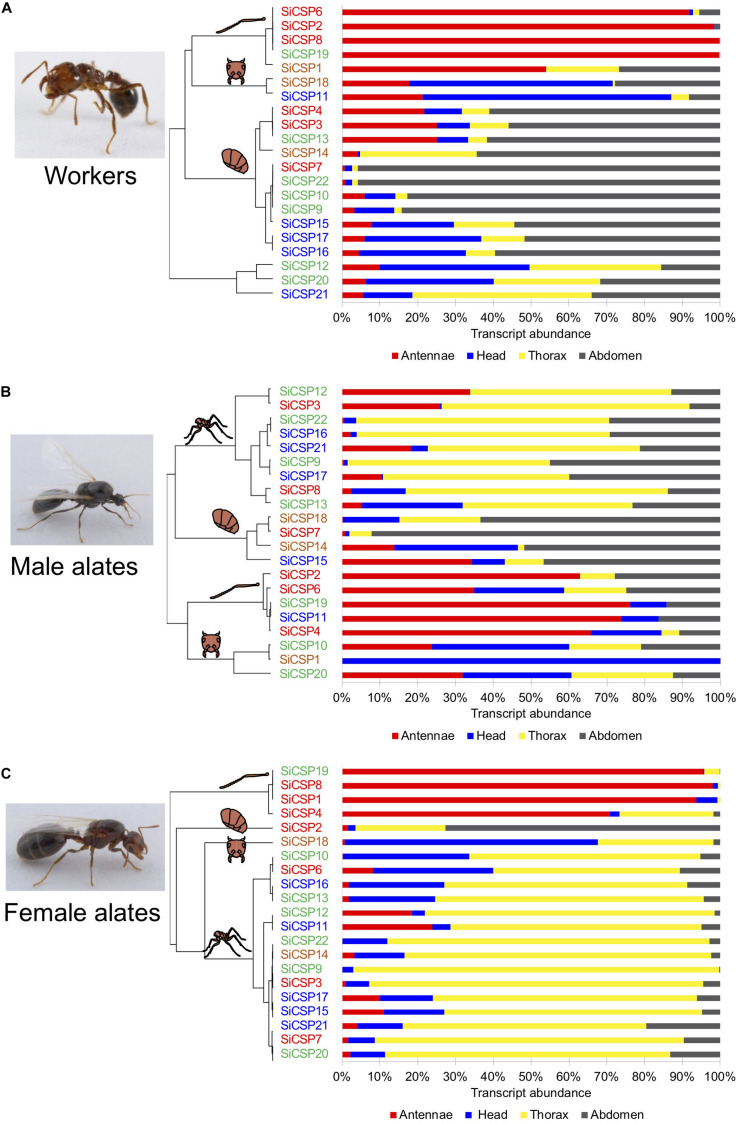
*SiCSP* relative expression distribution across tissues in **(A)** workers, **(B)** male, and **(C)** female alates. The percent of individual *SiCSP* expression in each tissue was calculated as follows: [*SiCSP* expression in tissue]/[total *SiCSP* expression across all four tissues] × 100. Data are representative of at least two independent preparations. Clusters indicate antenna-, head-, thorax-, and abdomen- biased *SiCSPs*. *SiCSPs* are color coded to match their phylogenetic relation.

**FIGURE 4:**
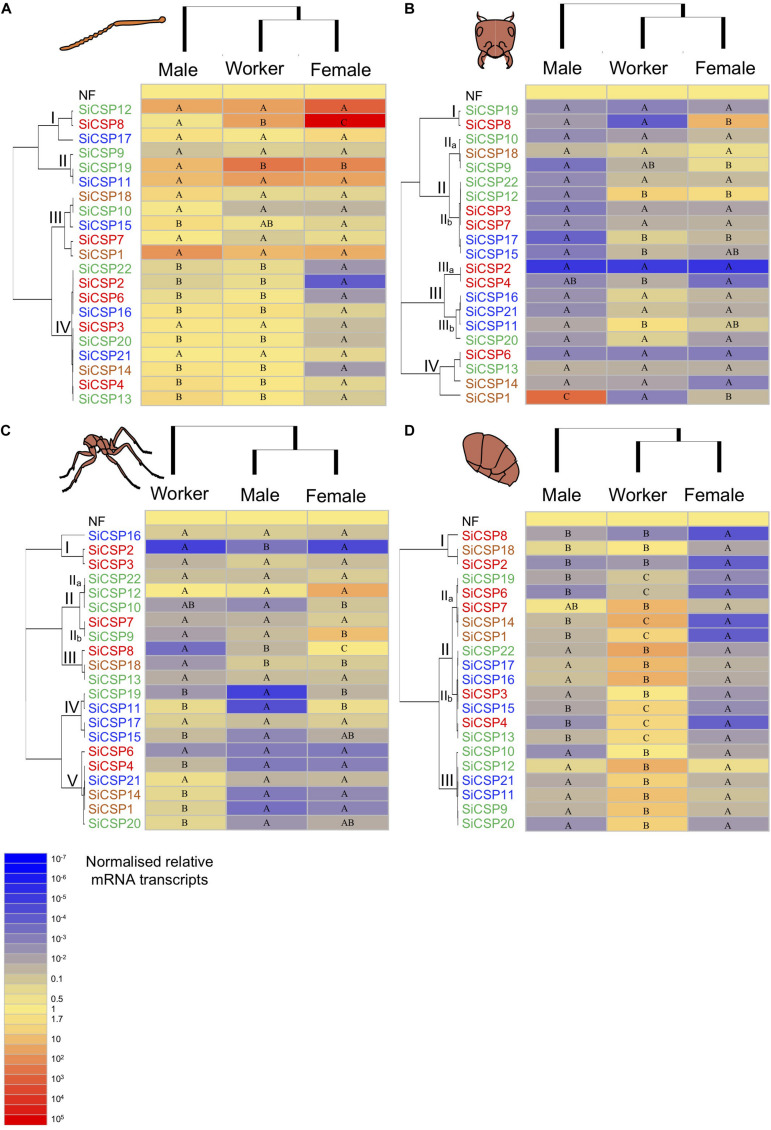
Expression of *SiCSPs* in fire ant workers, males and female alates. **(A)** Normalized *SiCSP* expression levels in the worker, male and female antennae. **(B)** Normalized *SiCSP* expression levels in the worker, male and female head. **(C)** Normalized *SiCSP* expression levels in the worker, male and female thorax. **(D)** Normalized *SiCSP* expression levels in the worker, male and female abdomen. Data are representative of at least three independent preparations. Clusters indicate co-expression networks of *SiCSPs* across the examined tissues. Within each row (i.e., *SiCSP*), different letters indicate significant differences at *P* < 0.05. The following terms are broadly used to describe the results in terms of number of normalized relative mRNA transcripts (NRTs): very low expression ≤10^–4^ to 10^–7^ NRTs, slightly low to low ≤10^–2^ to >10^–4^ NRTs, moderately robust <1 to >10^–2^ NRTs, robust to very robust ≥1 to <10 NRTs, slightly high to high expression ≥10 to <10^3^ NRTs, and very high expression ≥10^3^ to 10^5^ NRTs.

**FIGURE 5:**
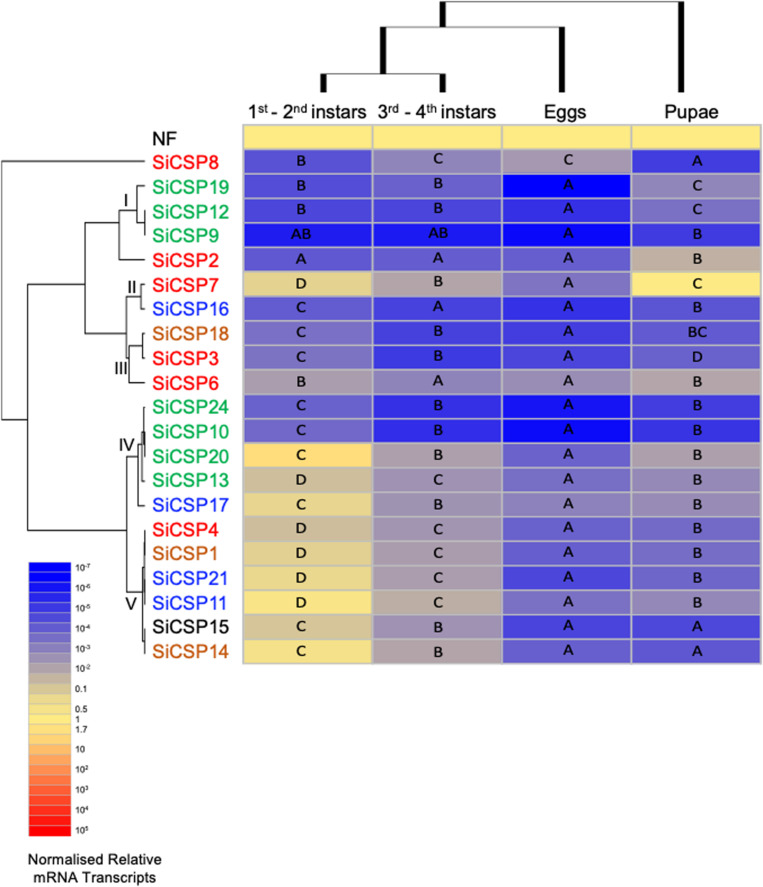
Expression of *SiCSPs* in fire ant eggs, larvae and pupae. Normalized *SiCSP* expression levels in 1st–2nd instar larvae, 3rd–4th instar larvae, eggs and pupae. Data are representative of at least two independent preparations. Clusters indicate co-expression networks of *SiCSPs* across the examined tissues. Within each row (i.e., *SiCSP*), different letters indicate significant differences at *P* < 0.05. The following terms are broadly used to describe the results in terms of number of normalized relative mRNA transcripts (NRTs): very low expression ≤10^–4^ to 10^–7^ NRTs, slightly low to low ≤10^–2^ to >10^–4^ NRTs, moderately robust <1 to >10^–2^ NRTs, robust to very robust ≥1 to <10 NRTs, slightly high to high expression ≥10 to <10^3^ NRTs, and very high expression ≥10^3^ to 10^5^ NRTs.

In order to obtain a clearer picture of the expression patterns of the *SiCSPs*, two separate analyses are presented for the adult ants: (A) the absolute number of transcripts as the fractions in each tissue relative to the total concentration of each *SiCSP* across all of the examined tissues (Figure 3) and (B) the normalized relative transcript abundance (with respect to the geometric mean of *EF1α* and *GAPDH* expression levels) (Figure 4). The former analysis gives the relative tissue distribution of each *SiCSP* and is a reflection of *SiCSP* absolute abundance in each tissue relative to the total concentration of the *SiCSP* in workers, female, or male alates. For the developmental stages only (B) the normalized relative transcript abundance in the four examined developmental stages (eggs, 1st–2st instar larvae, 3rd–4th instar larvae, and pupae) are shown (Figure 5).

#### Relative Tissue Distribution of *S. invicta* Chemosensory Proteins in Workers, and Male and Female Alates

Analyses of the tissue distributions of the *SiCSPs*, i.e., $\frac{{\left[SICSPX\right]}_{numberoftranscriptsperugofRNAinspecifictissue}}{{\left[SICSPX\right]}_{totalnumberoftranscrpitsperugofRNAacrossalltissues}}$, coupled with hierarchical clustering analyses allowed for the identification of groups of *SiCSPs* with similar patterns of transcript abundance across tissues in workers, males and female alates (Figure 3). These analyses also revealed that some CSPs appeared to be preferentially expressed in some tissues, with these tissue-biased expression patterns differing across castes and being somewhat phylogenetically related (Figure 3). *SiCSPs* preferentially expressed in the antennae of workers, males and females tended to belong to the group of ‘general CSPs’ (boxed in red in Figure 1), except for *SiCSP19* in workers, males and females, and *SiCSP11* in males, which were members of the *S. invicta* expansions (green and blue boxes in Figure 1). On the other hand, *SiCSPs* exclusively found in *S. invicta* (green and blue boxes in Figure 1) were seen most abundant in the worker abdomen and the male and female thorax.

In workers, *SiCSP* expression tended to be higher in the antennae and abdomen than in the head and the thorax (Figure 3A). *SiCSPs 6*, *2*, *8* and *19* were found almost exclusively in the worker antennae (>91.7% abundance), with *SiCSP1* also mainly distributed in the antennae but in lesser amounts (53.8%). In worker ants, abdomen-biased *SiCSPs* included *4*, *3*, *13*, *14*, *7*, *22*, *10*, *9*, *15*, *17* and *16* (56.0–95.8% abundance). *SiCSP18* and *11* were seen most abundant in the worker head, while no thorax-biased *SiCSPs* were found in workers. Notably, *SiCSP 12*, *20* and *21* showed comparable transcript abundance across all the examined tissues in workers.

To a lesser extent, the tissue distribution of *SiCSPs* in males mainly favored thorax tissues although subsets of antenna-biased, head-biased and abdomen-biased *SiCSPs* were also identified in males (Figure 3B). Transcript abundance in the male thorax of *SiCSPs 12, 3, 22, 16, 21, 9, 17, 8* and *13* varied between 44.7% and 66.8%. *SiCSPs 18, 7, 14* and *15* exhibited preferences for the male abdomen (46.2%–92.2% abundance), while *SiCSPs 2, 6, 19, 11* and *4* appeared most found in the male antennae, with transcript abundance between 34.9% and 76.2%. Conversely, *SiCSPs 20, 1* and *10* were most expressed in the head, with *SiCSP1* found almost solely in the male head (99.9% abundance).

Similar to males, the fractional tissue distribution of the *SiCSPs* in females showed a clear preference for the thorax, with 15 out of 21 *SiCSPs* being thorax-biased (Figure 3C). Notable exceptions to this were *SiCSPs 19, 8, 1* and *4*, that were preferentially found in the female antennae, showing transcript abundance between 70.8 and 98.0%. In addition, *SiCSP8* was found mainly in the head (66.9% abundance), and *SiCSP2* was found mostly in the female abdomen (72.6% abundance).

#### Chemosensory Protein Expression in Antennae of *S. invicta* Workers, and Male and Female Alates

In order to better present the results, the following terms are broadly used to describe the results in terms of number of normalized relative mRNA transcripts (NRTs): *very low* expression ≤10^–4^ to 10^–7^ NRTs, *slightly low to low* ≤10^–2^ to >10^–4^ NRTs, *moderately robust* <1 to >10^–2^ NRTs, *robust to very robust* ≥1 to <10 NRTs, *slightly high to high expression* ≥10 to <10^3^ NRTs, and *very high expression* ≥10^3^ to 10^5^ NRTs. Overall *SiCSP* expression in the antennae across workers, males and females ranged between robust and very high, with the exception of some *SiCSPs* in female antennae whose expression varied between slightly low and low (Figure 4A). Hierarchical clustering by caste (i.e., columns) revealed that the overall *SiCSP* expression in workers was more similar to female alates than to males, while hierarchical clustering by *SiCSP* (rows) yielded four antennal co-expression clusters, namely groupings of CSPs with similar expression patterns. These clusters of co-expressed genes consisted of subgroups of *SiCSPs* with no apparent phylogenetic relationship: I) *SiCSPs 12, 8* and *17*, II) *SiCSPs 9, 19* and *11*, III) *SiCSPs 18, 10, 15, 7* and *1*, and IV) *SiCSPs 22, 2, 6, 16, 3, 20, 21, 14, 4*, and *13* (Figure 4A). In cluster I, *SiCSP12* showed high expression in males and workers and very high expression in female alates, although these differences were not statistically significant. *SiCSP8*, which was differentially expressed across castes, displayed robust expression in males, high expression in workers and very high expression in female alates. On the other hand, *SiCSP17* exhibited similar expression levels among castes although expression of this gene was lower than that seen for *SiCSPs 12* and *8*.

Within the antennal co-expression cluster II, *SiCSP19* and *11* were equally expressed across caste, although *SiCSP11* showed overall greater expression (i.e., high) than *SiCSP19*, which was only robustly expressed in adult antennae. Expression of *SiCSP19* in the antennae varied significantly between males and workers and males and females alates, ranging between slightly high (males) and high (workers and female alates). Expression levels in the antennal co-expression cluster III fluctuated between slightly low to very high. However, expression values within the same *SiCSP* remained not statistically distinct among male, worker and female antennae for the majority of the *SiCSPs* in grouping III (i.e., *SiCSPs 18, 10, 7* and *1*). Only *SiCSP15*, which was high to very highly expressed in adult antennae, showed significantly different expression among castes. Antennal co-expression cluster IV included ten *SiCSPs* which included members of the general, ant expansion and *S. invicta*-specific *CSPs*. *SiCSP* expression in male and worker antennae within grouping IV was overall robust, while the expression of *SiCSPs 22, 2, 6, 16, 20, 14, 4* and *13* in female antennae varied between low (*SiCSP2*) and slightly robust (*SiCSPs 16* and *4*). Within this group of co-expressed genes, only *SiCSPs 3* and *21* showed no significant differences among castes, while the rest of the *SiCSPs* within grouping IV was significantly lower expressed in female antennae than in male and worker antennae.

#### Chemosensory Protein Expression in the Head of *S. invicta* Workers, and Male and Female Alates

In general, expression of the *S. invicta CSPs* in the adult head was lower than in adult antennae and was similar between workers and female alates. Furthermore, the overall expression of *SiCSPs* in the male head was somewhat lower than in the other two examined castes (Figure 4B). Hierarchical clustering generated five clusters of co-expressed *SiCSPs* which included subgroups of phylogenetically similar and more distant *SiCSP*s. Co-expression cluster I incorporated *SiCSPs 19* and *8*, which differed in expression pattern across castes. *SiCSP19* expression was slightly low in the head of all the studied castes, whereas *SiCSP8* expression was low in the male and worker head, with no statistical differences seen between the two tissues. However, *SiCSP8* was significantly much more highly expressed in the head of female alates as compared to the other two samples.

Co-expression cluster II encompassed two sub-clusters, II_a_ (*SiCSPs 10, 18* and *9*) and II_b_ (*SiCSPs 22, 12, 3, 7, 17* and *15*). Within sub-cluster II_a_, *SiCSP 10* and *18* expression levels were low to robust, respectively, and did not exhibit significant differences among castes. Conversely, SiCSP9 exhibited robust expression in the female head, which was statistically higher (*P* < 0.05) than in the male head. Expression levels among the *SiCSPs* from sub-cluster II_b_ varied between low and slightly high. This sub-cluster comprised general *SiCSPs* (i.e., *SiCSPs 3* and *7* boxed in red in Figure 1) and *S. invicta*-specific *SiCSPs* (*SiCSPs 22* and *12* - boxed in green in Figure 1 – and *SiCSPs 17* and *15* – boxed in blue in Figure 1) which, in turn, displayed similar co-expression patterns between them. *SiCSP22* expression was low in the head of all ant castes, and *SiCSP12* expression was significantly lower (*P* < 0.05) in the male head than in worker and female heads. Expression levels of *SiCSPs 3* and *7* was low across adult heads, with no significant differences among castes. Similar to *SiCSP12*, the expression of *SiCSP17* and *15* was statistically lower (*P* < 0.05) in male heads than in worker and female heads. Significant differences were observed between female and male heads in the expression of *SiCSP17*, but not in *SiCSP15*.

Similar to co-expression cluster II, co-expression cluster III could be divided into two sub-clusters that were phylogenetically related. Sub-cluster III_a_ included *SiCSP2* and *4* (general CSPs – boxed in red in Figure 1) whose expression levels ranged from very low (*SiCSP2* – worker, male and female heads) to slightly low (*SiCSP4* – worker head). Significant differences in expression were seen in *SiCSP4* between worker (higher) and male/female heads (lower). Sub-cluster III_b_ contained only *SiCSPs* found exclusively in *S. invicta* (i.e., *SiCSPs 16*, *21*, and *11* – boxed in blue in Figure 1 and *SiCSP 20* – boxed in green in Figure 1). Within this sub-cluster, the expression of the majority of the *SiCSPs* (i.e., *SiCSP16*, *21*, and *20*) was in general not statistically different and low across worker, male and female heads, with the exception of *SiCSP11* which was somewhat robust expressed in worker and female heads and significantly higher (*P* < 0.05) in these two tissues than in the male head.

One outlier, namely *SiCSP1*, was found within co-expression cluster IV. *SiCSP1* was differentially expressed among castes and was very highly expressed in the male head. It showed slightly low expression in the female head, and low expression in worker heads. Conversely, the other members of co-expression cluster IV (*SiCSP6*, *13*, and *14*) were expressed at somewhat low levels and did not show statistical differences across castes.

#### Chemosensory Protein Expression in the Thorax of *S. invicta* Workers, and Male and Female Alates

For the most part, *SiCSP* expression in worker, male and female thoraces ranged between very low to robust, with the exception of *SiCSPs 22* and *9* which were highly expressed in the female thorax (Figure 4C). Unlike for the rest of the tissues examined (i.e., antennae, head, and abdomen), the *SiCSP* expression profile of adult thoraces was more similar between males and females than between workers and both reproductive castes, as revealed by the hierarchical clustering analysis. Clustering by *SiCSPs* generated five co-expression clusters that included sub-clusters of phylogenetically related and distant CSPs sharing similar expression profiles across castes: (I) *SiCSPs 2*, *3*, and *16*, (II) *SiCSPs 22*, *12*, *10*, *9* and *7*, (III) *SiCSPs 8*, *18* and *13*, (IV) *SiCSPs 13*, *19, 11*, *17*, *15*, and (V) *SiCSPs 6*, *4*, *21*, *14*, *1*, *20* (Figure 4C).

Expression of *SiCSP2* in the thorax varied between very low in workers and females to low in males. The expression of *SiCSP3* in the thoraces of the three castes was slightly robust and, although *SiCSP3* followed the trend of *SiCSP2*, being lower in workers and females than in male, these differences were not found to be statistically significant. *SiCSP16* was the outlier in co-expression cluster II, showing similar slightly robust expression across adult thoraces with no statistical differences. Co-expression cluster II consisted of two sub-clusters: II_a_ (*SiCSPs 22, 12* and *10* – formed by *SiCSPs* found exclusively in *S. invicta*, which are boxed in green in Figure 1) and II_b_ (*SiCSps 7* and *9*). Within sub-cluster II_a_, *SiCSPs* expression fluctuated between low and somewhat high. Among worker, male and female thoraces, no significance differences in expression were seen in *SiCSP22* and *12*, which exhibited slightly robust and somewhat high expression respectively in adult thoraces. *SiCSP10*, however, was significantly lower expressed in males than females but not in workers, showing low expression in the male and worker thoraces and slightly robust expression in the female thorax. *SiCSP7* and *9* appeared to be more expressed in the female thorax (slightly robust and high, respectively) than in the worker and male thoraces (slightly low), although only *SiCSP9* was found to be significantly (*P* < 0.05) higher expressed in the female head.

The *SiCSPs* included in co-expression cluster III were not phylogenetically related, and their expression varied between low to robust. *SiCSP8*, whose expression in the thorax was low in the males, slightly robust in workers and robust in females, was statically differentially expressed across castes. *SiCSP18* in the thorax was low expressed in workers and somewhat robustly expressed in males and females and only showed statistical differences between workers and males and workers and females. The expression of *SiCSP13* in adult thoraces was slightly robust, and no significant differences were seen among castes. Co-expression cluster IV consisted of members of the two *S. invicta* expansions (i.e., *SiCSP19* – colored in green in Figure 1 – and *SiCSPs 11*, *17*, and *15* – colored in blue in Figure 1). *SiCSPs 19* and *11* were expressed at very low levels in the male thorax and were significantly more highly expressed in the thoraces of workers and females. *SiCSP17* was somewhat robustly expressed throughout adult thoraces and showed no differential expression. In contrast, *SiCSP15* was differentially expressed in the male thorax and exhibited low expression, as opposed to workers and females which showed moderate low expression. *SiCSPs 6*, *4*, *21*, *14*, and *20* formed co-expression cluster V and mainly appeared to be more highly expressed in the worker thorax than in male and female thoraces. The expression of *SiCSPs 4*, *14*, *1*, and *20* was statistically higher (*P* < 0.05) in the worker thorax than in the thoraces of males and females, exhibiting moderate low (e.g., *SiCSP4*) and slightly robust (e.g., *SiCSPs 14*, *1* and *20*) expression. *SiCSPs 6* and *21* showed low to slightly robust expression, respectively, in adult thoraces with no statistical differences found among castes.

#### Chemosensory Protein Expression in the Abdomen of *S. invicta* Workers, and Male and Female Alates

Overall SiCSP expression in the abdomens of *S. invicta* was slightly low in males and females and robust to high in workers (Figure 4D). Despite some apparent differences in expression, the hierarchical clustering analysis exposed similar co-expression profiles between worker and female abdomens, with a more distant expression profile seen for the male abdomen. Three groupings or clusters of co-expressed *CSPs* were inferred by hierarchical clustering, showing phylogenetic relatedness: (I) *SiCSPs 18*, *2*, and *8*, (II) *SiCSPs 19*, *6*, *7*, *14*, *1*, *22*, *17*, *16*, *3*, *15*, *4*, *13*, and (III) *SiCSPs 10*, *12*, *21*, *11*, *9*, and *20* (Figure 4D). *SiCSPs 18*, *2* and *8*, in co-expression cluster I, showed significantly lower expression in the female abdomen than in worker and male abdomens. *SiCSP8* and *2* expression was low in worker and male abdomens, and very low in the female abdomen, while *SiCSP18* was robustly expressed in workers and males and showed low expression in the female abdomen.

Co-expression cluster II consisted of two sub-cluster, II_a_ (*SiCSPs 19*, *6*, *7*, *14* and *1*) and II_b_ (*SiCSPs 22*, *17*, *16*, *3*, *15*, *4* and *13*). Within sub-cluster II_a_, *SiCSPs 19*, *6*, *14* and *1* exhibited significant (*P* < 0.05) differential expression across all examined castes, with *SiCSP7* showing significant differential expression between the worker abdomen and the female abdomen. *SiCSPs 19*, *14* and *1* showed moderately low expression in the abdomen of males, with high (e.g., *SiCSP19*) to very high expression (e.g., *SiCSPs 14* and *1*) in the abdomen of workers but lower expression in the abdomen of female alates. SiCSP7 displayed moderately low expression in the female abdomen with higher expression in male and worker abdomen. Among the examined abdomen, *SiCSP6* expression was somewhat robust in workers, slightly low in males and low in females. In sub-cluster II_b_, the expression of *SiCSP15*, *4* and *13* in the abdomen showed significant differences across caste, while the expression of *SiCSPs 22*, *17*, *16* and *3* only presented differences between the worker and male abdomen and between the worker and female abdomen. *SiCSP15* and *13* exhibited moderately low expression in males, low expression in females but high expression in workers. Similarly, *SiCSP4* also showed high expression in the worker abdomen, moderately low expression in males, and low expression in females, although its expression in male and female abdomen was much lower than that seen for *SiCSPs 15* and *13*. Comparatively, *SiCSPs 22*, *17*, and *16* displayed quasi-identical co-expression pattern across castes, with moderately low expression in the abdomen of males and females and high expression in worker abdomen. *SiCSP3* also appeared more highly expressed in worker abdomen than in male and female abdomen.

Co-expression cluster III included only members of the two *S. invicta* expansions (i.e., *SiCSPs 10, 12, 9* and *20* – boxed in green in Figure 1 – and *SiCSP21* and *11* – boxed in blue in Figure 1). All the *SiCSPs* within this co-expression cluster showed significantly (*P* < 0.05) higher levels of expression in the worker abdomen than in the male and female abdomen. *SiCSP10* displayed robust expression in the worker abdomen and low expression in the male and female abdomen. Expression of *SiCSP12* in the abdomen was much higher across the examined castes than that seen for *SiCSP10*, with robust levels of expression in males and females and high expression in workers. *SiCSPs 21, 11*, and *9* showed very similar expression patterns across adult abdomen samples, namely high in worker abdomen and somewhat robust in male and female abdomen. *SiCSP20* was also highly expressed in the worker abdomen but, unlike *SiCSPs 21, 11* and *9, SiCSP20* appeared low expressed in the male and female abdomens.

#### Chemosensory Protein Expression in *S. invicta* Developmental Stages

*SiCSP* expression was examined in *S. invicta* eggs, larvae, and pupae (Figure 5). Larvae were separated into 1st/2nd (early) and 3rd/4th (late) instars based on size and morphology. One-way ANOVA yielded significant differential expression of the *SiCSPs* within each developmental stage examined (*P* < 0.001). Overall, expression of *SiCSPs* was low within all the developmental stages examined. Hierarchical clustering analysis by column (i.e., developmental phase) revealed similar co-expression patterns between early- and late-instar larvae, and a much distant and reduced *SiCSP* expression profiles in pupae and eggs (Figure 5).

In early-instar larvae, *SiCSPs 14*, *11* and *20* showed somewhat high expression, *SiCSPs 21*, *17*, *7*, *1*, *15*, *13*, and *4* robust to slightly robust expression, while the rest of the *SiCSPs* (*SiCSPs* 6, 3, 18, 10, 22, 16, 2, 8, 19, 12, 9) exhibited low to very low expression (Supplementary Figure S1). Although early- and late-instar larvae showed similar expression profiles, overall *SiCSP* expression in late-instars was much lower than in early instars, with all *SiCSPs* exhibiting slightly low to very low abundance of normalized transcripts. Late-instar larvae displayed low expression of *SiCSPs 11*, *14*, *7*, *20*, *21*, *1*, *4*, *17*,*15*, *13*, *8*, and *6*, and low to very low expression of the remaining *SiCSPs* (*SiCSP19*, *2*, *12*, *16*, *18*, *3*, *22*, *10*, *9*). In eggs, SiCSP expression was low to very low, while *SiCSP* expression levels appeared to increase for pupae, which showed significant differential expression that ranged between robust (*SiCS7*) to very low (*SiCSP10*) (Supplementary Figure S1).

One-way ANOVA analysis of the normalized expression data showed that *S. invicta CSPs* are significantly (*P* < 0.001) differentially expressed across developmental stages (Figure 5). Hierarchical clustering by *SiCSP* allowed for the identification of five co-expression CSP clusters: (I) *SiCSPs 19*, *12* and *9*, (II) *SiCSPs 7* and *16*, (III) *SiCSPs 18*, *3* and *6*, (IV) *SiCSPs 22*, *10*, *20*, *13* and *17*, and (V) *SiCSPs 4*, *1*, *21*, *11*, *15* and *14*. These groupings of co-expressed *CSPs* revealed shared expression within more closely related SiCSPs, but also cases among more distantly related SiCSPs (Figure 5). The expression patterns of *SiCSP8* and *2* across developmental stages were not shared with the other *SiCSPs*. *SiCSP8* expression was low in early-instar larvae and pupae and moderately low in late-instar larvae and egg, with significance differences found between both larval stages, early-instar larvae and eggs, and pupae and eggs. *SiCSP2*, on the other hand, was more highly expressed in pupae than in the other developmental phases, which showed no significant differences among them.

Within co-expression cluster I, expression of *SiCSPs 19, 12* and *9* (members of one of the *S. invicta* expansions boxed in green in Figure 1) ranged from low to very low among developmental stages, with expression levels of *SiCSP19* being statistically different across all developmental stages, and expression of *SiCSP12* being not statistically different between larvae (early and late instars) but distinct when comparing both larval stages (early and late instars) to pupae, and larvae (early and late instars) to eggs, and pupae to eggs. The expression of *SiCSP9* was very low and similar across all the developmental stages.

Expression of *SiCSPs 7* and *16* (co-expression cluster II) in developmental stages varied between very low (*SiCSP16* in late-instar larvae and eggs) to robust (*SiCSP7* in pupae). Significant different levels of expression were observed in *SiCSP7* across all the examined developmental stages, showing more robust expression in early-instar larvae and pupae than in late-instar larvae and eggs. *SiCSP16* displayed low expression in early-instar larvae and pupae and very low expression in late-instar larvae and eggs. While no significant differences in expression levels of *SiCSP16* were noticed among early and late larval instars and pupae and late larval instars and eggs, significant differences were detected between both larval instar and between early-instar larvae and eggs.

Within co-expression cluster III (i.e., *SiCSPs 18, 3* and *6*) expression levels ranged from moderately low to very low. *SiCSPs 18* and *3* displayed similar expression profiles, with very low expression in late-instar larvae and eggs, and low expression in early-instar larvae and pupae. Significantly different levels of expression were observed for *SiCSPs 18* and *3* between both larval stages and late-instar larvae and pupae. In addition, a significant increase of expression was observed between early-instar larvae and pupae for *SiCSP18* but not for *SiCSP3*. *SiCSP6* showed a comparable trend of expression to *SiCSPs 18* and *3*, although with slightly higher expression values. Also, *SiCSP6* displayed low levels of expression throughout all developmental stages, although expression was significantly lower in late-instar larvae and eggs than in the other two phases.

Co-expression cluster IV, which includes *SiCSPs 22, 10, 20, 13* (members of one of the *S. invicta* expansion boxed in green in Figure 1) and *17* (member of the *S. invicta* expansion boxed in blue in Figure 1), displayed somewhat high to very low expression values. *SiCSP22* and *10* showed almost identical expression profiles across developmental stages, with lower expression in eggs, pupae, and late-instar larvae than in early-instar larvae. In addition, significant differences were seen comparing eggs to pupae, and eggs to both larval stages. *SiCSP20* was slightly highly expressed in early-instar larvae and showed low expression in late-instar larvae, pupae and eggs. Expression of *SiCSP20* was significantly lower in eggs, late-instar larvae and pupae than in early-instar larvae. *SiCSP13* and *17* showed a similar trend as *SiCSP20*, with higher levels of expression in early-instar larvae and much (significantly) as compared to lower expression levels seen in eggs, late-instar larvae, and pupae.

Co-expression cluster V, comprising *SiCSPs 11, 21, 15* (members of the *S. invicta* expansion boxed in blue in Figure 1), *1, 4* and *14*, exhibited similarities with cluster IV since the overall expression of this group of *SiCSPs* was robust in early-instar larvae but low/very low in the other examined stages. *SiCSP4, 1*, and *21* were statistically differentially expressed (*P* < 0.05) across all developmental phases, whereas *SiCSP11, 15* and *14* expression levels were statistically different (*P* < 0.05) comparing both larval stages (early to late instars), larvae to eggs, and larvae to pupae, and not significantly different when comparing eggs to pupae.

### Protein Motifs Identified in the *S. invicta* CSPs

**FIGURE 6:**
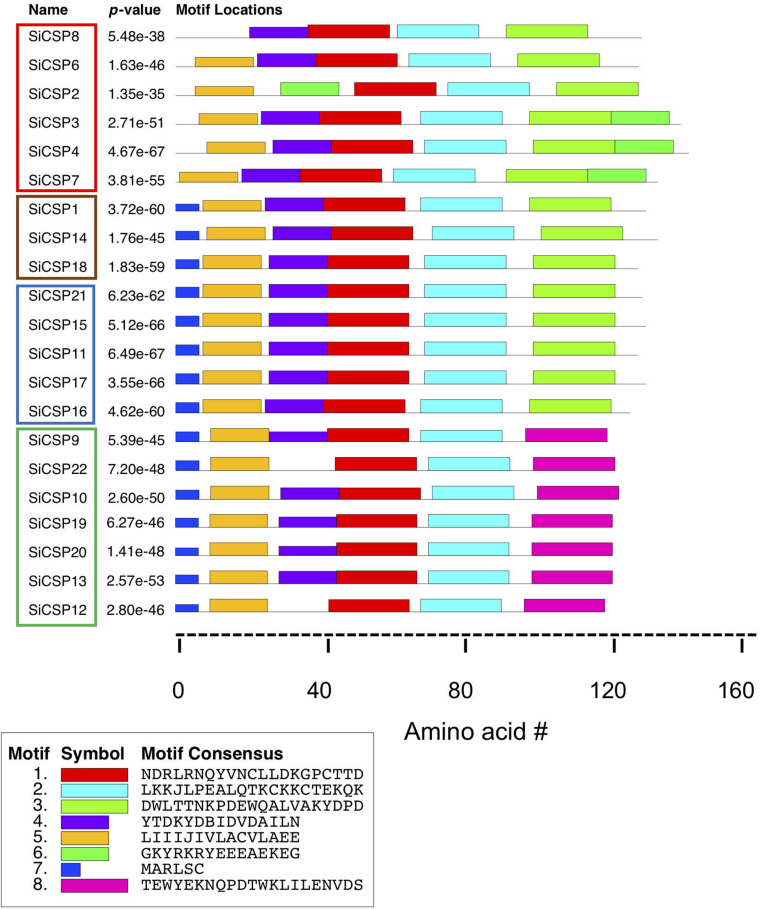
Motif analyses of *S. invicta* CSPs. Position of the 7 most common amino acid motifs identified in *S. invicta* and 71 more CSPs using the MEME server (version 4.11.2, http://meme-suite.org/tools/meme).

The MEME server (Bailey and Elkan, 1994) was used to identify conserved amino acid motifs present in the 21 *S. invicta* CSPs, along with the other homologs used to build the phylogenetic tree. Eight distinct motifs were identified with e-values < 1.2 e^–37^, and their distribution within *S. invicta* (Figure 6) and the 92 CSP set (Supplementary Figure S2) determined. Motifs 1 and 2 (red and turquoise, respectively) were found in all of the CSPs examined, including the full 21 SiCSPs, and contain the four conserved cysteines. Motif 3 (neon green), at the C-terminus, was also found in the majority of SiCSPs, with the exception of SiCSPs 10, 12, 13, 19, 20, 22, and 9 (*S. invicta* CSP expansion boxed in green in Figure 1) which contained a separate motif (8, in pink) instead, that appeared to be unique to *S. invicta* and mutually exclusive with motif 3 (Supplementary Figure S2). Motif 4 (purple) was also widely distributed within the majority of the CSPs examined. In *S. invicta*, motif 4 was missing in SiCSPs 12, 22, and 2, although in the latter, this motif appeared to be substituted by a shuffled motif 6 (green) (Figure 6). Motif 6 (green) was found as an extended C-terminus in SiCSPs 3, 4, and 7 but not in SiCSP2. Motif 5 and 7 (orange and dark blue, respectively) were found at the N-terminus of the CSPs and are both part of the signal peptide sequence. Motif 5 was found in most of the CSPs, with only few exceptions (e.g., SiCSP8), while Motif 7 only appeared in the CSPs that formed part of the CSP ant expansion (Supplementary Figure S2, CSP ant expansion illustrated within the gray shaded area in Figure 1).

## Discussion

Chemosensory proteins represent one branch of at least three phylogenetically un-related insect protein families, that are however, considered partially functionally related. The other two members are the arthropod OBPs and the lipid transporters classified as Niemann-Pick type C_2_ proteins (NPC_2_) (Pelosi et al., 2014). All three classes of proteins are small (∼12–20 kDa), soluble, ligand-binding proteins, that are often considered capable of solubilizing hydrophobic compounds including odorants, volatiles, and lipids within the aqueous milieu of the organism and/or cell. These molecules can encompass pheromones, semio-chemicals, hydrocarbons, fatty acids, amongst other hydrophobic compounds, but can also potentially include soluble substrates such as carbohydrates and other molecules. In arthropods, CSPs exist as multigene families, and have been associated with chemoreception of both olfactory and gustatory cues. Aside from numerous studies showing expression of CSPs in antennae (for recent reports see Antony et al., 2016; Hu et al., 2016; Xue et al., 2016; Jing et al., 2020), these proteins have also been found in many other tissues some of which contain specialized sensilla, e.g., in tarsal chemosensilla that respond to non-volatile surface chemicals and are involved in gustatory detection (Ma et al., 2016). In the latter case, transcriptomic analyses in the tea geometrid, *Ectropis obliqua*, revealed sex and leg-specific expression of CSPs, suggesting their involvement in taste detection. From these and other studies, it is now well established that many CSPs are expressed outside olfactory organs, functioning in chemosensory (e.g., taste) and non-chemosensory roles (e.g., development). Transcriptomic analyses in several ant species revealed that many OBPs and CSPs are expressed primarily in non-olfactory tissues (McKenzie et al., 2014), strongly suggesting non-olfactory roles for many of these proteins in ants. In *A. mellifera*, *AmCSP5* is not expressed in the antennae, but instead has been implicated in embryonic development, with high expression noted in queen ovaries and eggs (Maleszka et al., 2007). In addition, proteomic analyses have indicated the presence of CSPs in insect hemolymph (Hou et al., 2016).

Our data indicate caste-specific expression patterns of the *S. invicta* CSPs. Transcripts for most of the *SiCSPs* could be detected at robust and high levels in worker and male antennae. Levels of *SiCSP* expression in female alate antennae were notably lower, with the expression of at least four *SiCSPs*, i.e., *22*, *2*, *6*, and *14*, being low in female alate antennae but robustly expressed in workers and male alate antennae. This suggests that in *S. invicta*, similar to *H. saltator*, olfactory responses depend on reproductive status (Ghaninia et al., 2017). Pheromonal inhibition by the reproductive queen(s) and/or ecological adaptations - since female alates remain most of the time in the nest and are not involved in colony maintenance – might account for *S. invicta* female alates (i.e., virgin females) being less responsive to olfactory cues than male alates and workers. Generalized robust expression of *CSPs* in male alates, on the other hand, might reflect the need for male alates to respond to olfactory signals in the context of mating (Ghaninia et al., 2018).

In workers, *SiCSP19* was the most highly expressed *CSP*, consistent with its original discovery in *S. invicta* antennae (Leal and Ishida, 2008; Gonzalez et al., 2009). The protein product of *SiCSP19* has been localized to the tip (A9-A10 segments) of the antennal club (Leal and Ishida, 2008). As the porous sensilla contain self-enclosed neuronal compartments located in the *S. invicta* antennal club that are not contiguous with the cuticle and hemolymph of the rest of the antennae (Renthal et al., 2003), it was concluded that SiCSP19 is most likely found in the olfactory sensilla (Gonzalez et al., 2009). Intriguingly, SiCSP19 is not an ortholog of the *C. japonicus* CSP1 (CjCSP1) that has been shown to be localized to chemosensory sensilla and implicated as critical for binding of hydrocarbons implicated in nestmate/non-nestmate discrimination (Ozaki et al., 2005). The ortholog of *CjCSP1* appears to be *SiCSP1*, which, while equally highly expressed across female alate, male and worker antennae, is more preferentially expressed in the male head. Proteomic analyses of *S. invicta* worker antennae also revealed the presence of SiCSP12 (Gonzalez et al., 2009), that was not only highly expressed in worker antennae in our dataset, but also in male and female alate antennae. In addition, robust to high expression of *SiCSPs 8* and *11* were noted in worker, male and female alate antennae. Whether these proteins (and/or other SiCSPs) are made in the antennae will require further deeper probing of the proteomic content of these structures.

A limited number of downstream behavioral effects have been attributed to CSPs involved in odorant detection. The *B. dorsalis BdorCSP2* has been implicated in insect responses to rhodojaponin-III, a non-volatile diterpene which shows antifeedant and anti-ovipositing activities against insects (Yi et al., 2013). BdorCSP2 was shown to be capable of binding rhodojaponin-III *in vitro*, and RNAi mediated knockdown of *BdorCSP2* decreased the effects of the chemical compound on the insect. *SiCSP4* is the ortholog of *BdorCSP2* and is robustly expressed in the antennae of fire ant males and workers and in much higher levels in the worker abdomen. Several CSPs, highly expressed in *A. cerana* antennae, have been shown to bind various floral chemicals (Li H.L. et al., 2016) and two CSPs in the mite *Tyrophagus putrescentiae* have been implicated in host recognition (Li X.M. et al., 2016), although these studies lack experimental designs that would directly test the role(s) of these CSPs in mediating specific behaviors. Differential male and female-specific expression of olfaction genes, has been reported in a number of insects including the oriental fruit fly (*Bactrocera dorsalis*) and the hover fly *Scaeva pyrastri* (Li X.M. et al., 2016; Liu et al., 2016). Caste and sex-specific expression of chemosensory genes has also been reported in termites and honeybees (Mitaka et al., 2016; Jain and Brockmann, 2020). When the absolute number of *SiCSP* transcripts is examined by tissue distribution, clear caste- and sex-specific differences are apparent. In workers, *SiCSP* transcripts are distributed mainly in the antennae and the abdomen. This suggests these SiCSPs may function as ligand carriers in antennal chemosensation, and that in the abdomen these proteins may act to bind/sequester endogenous hydrophobic compounds that can include pheromones (for release), hydrocarbons and lipids (for delivery to the cuticle), and/or endocrine molecules (endogenous hormones and other compounds). In contrast, in female alates, *SiCSP* transcripts appear to be widely distributed in the thorax, and to a lesser extent, the male thorax appeared also enriched in *SiCSP* expression. These tissues include the legs and the wings that contain tarsal and wing chemosensory sensilla, some of which have gustatory roles (Fleischer and Krieger, 2018; He et al., 2019). As reveal by the clustering analyses, similar patterns of CSP expression in *S. invicta* male and female thoraces could indicate roles for these CSPs in gustatory and/or taste functions in this ant species. Gustatory pathways in *Drosophila* have been reported to be activated by pheromonal cuticular hydrocarbons (CHCs) (Fleischer and Krieger, 2018). In ants, select CHCs also mediate social interaction between colony members and can act as mating pheromones (Kather and Martin, 2015; Slone et al., 2017). In *Drosophila*, some pheromonal CHCs are primarily detected via taste organs on legs and wings (Fleischer and Krieger, 2018). It is intriguing to speculate that in male and female *S. invicta* alates, some *SiCSPs*, (particularly, *S. invicta*-specific CSPs) expressed in the thorax may function to bind mating CHCs, and that sensilla located in the male and female thorax participate in mate recognition.

*SiCSP* expression levels and tissue distribution appear to also reflect the task distribution of the various castes. Workers are “pluripotent” in task and are hence exposed to a significantly larger range of environmental compounds than female or male alates. Female alates need to mate, establish the colony, and subsequently act as the reproductive unit of the colony, and thus antennal chemosensation is likely to be far less important than in workers. In addition, queen pheromones act to regulate offspring behavior, but the queen herself may be largely non-responsive to these chemicals. Finally, male alates have only one task, and that is to mate. Therefore comparatively, in males, the requirements for and functions of the CSPs may be more limited, consistent with their lower expression and scattered tissue distribution.

A lack of congruent patterns expression of orthologous CSPs was generally seen when our data are compared to CSP expression analyses in *C. japonicus*. For example, the most highly expressed CSPs in *C. japonicus* were *CjCSPs 1*, *3*, *7*, *12*, and *13*, with only *CjCSPs1*, *3*, and *7* having true orthologs in *S. invicta* (Hojo et al., 2015). Of the corresponding *SiCSPs* only *SiCSP1* was highly expressed in worker antennae. These data suggest little cross-species conservation of the expression patterns of orthologous CSPs.

In general, the *SiCSPs* were poorly expressed in the developmental stages examined and were especially low in eggs and pupae. Some expression was seen in the various larval stages, and when compared across the developmental stages, *SiCSP* expression appeared to peak in 1st/2nd instar larvae. These data suggest the potential for a role for some *SiCSPs* as ligand carriers in larvae, potentially for distribution and delivery of cuticular hydrocarbons and lipids and/or of developmental hormones and other molecules. Apparent co-expression of some *CSPs* has been reported in the antennae of *C. japonicus* (Hojo et al., 2015). Hierarchical clustering analyses examining potential co-regulation of phylogenetically related *SiCSPs* revealed patterns of co-expression of *SiCSPs* expression within caste tissues and between developmental stages. However, notable divergences were also seen. No robust co-expression consistent with phylogeny was observed among *SiCSPs* across worker, male and female alate antennae. In contrast, in the head, thorax, abdomen and developmental stages co-expression of phylogenetic sub-groups could be discerned. Our data revealed potential examples of co-regulation of both divergent and convergent lines of *SiCSPs*.

These data, together with the characterization of the expression profiles of the *S. invicta* OBPs (Zhang et al., 2016), indicate that both OBPs and CSPs show discrete expression profiles within *S. invicta*. These profiles extended between workers, male and female alates, and across various developmental stages and supports an emerging model of these proteins as general ligand carriers involved in a wide range of physiological processes beyond olfaction and/or chemosensation. Comparison between *SiOBP* expression distribution and *SiCSP* expression revealed a number of interesting insights. Similar to the *SiCSPs*, only a subset of the *SiOBPs* were highly expressed in the worker, male and female alate antennae. However, *SiCSP* expression was more enriched in the antennae than *SiOBP* expression (Zhang et al., 2016). In the abdomen, *SiCSP* expression was enhanced in the worker but not in male and female alates, while *SiOBP* expression showed the opposite trend (more highly expressed in the male and female abdomen than in the worker abdomen). The fire ant head and thorax tissues showed lower expression of the *SiCSPs* than of the *SiOBPs*. In contrast, *SiCSP* expression was much higher than *SiOBP* expression in the various developmental stages (Zhang et al., 2016). These comparisons indicate a general inverse correlation between *SiCSP* and *SiOBP* expression. This may suggest compensatory functions and/or that these proteins might perform unique tasks within different tissues and, in some instance, may complement each other. Thus, the differential regulation of *SiCSPs* and *SiOBPs* may be linked to the substrates they may recognize.

Motif analyses of the SiCSPs revealed a core set of three motifs (i.e., motifs 4, 1, and 2) found in almost all of the SiCSPs as well as two slightly different conserved signal peptide sequences consisting on motif 5 alone and a combination of motifs 7 and 5. Additional motifs (i.e., 3, 6, and 8) were found at the C-terminal end in subsets of the SiCSPs. Essentially, three different C-terminal ends were observed in SiCSPs, which yielded three distinct groups of SiCSPs. Group I, which included one of the fire ant-specific CSP gene expansion (SiCSPs 9, 22, 10, 19, 20,13, and 12) and the shortest C-terminus, containing motif 8. Group II containing motif 3 and including SiCSPs 1, 14, 18 and the second fire ant-specific CSP gene expansion (i.e., SiCSPs 21, 15, 11, 17, and 16). And Group III (SiCSPs 8, 6, 2, 3, 4, and 7) with the longest C-terminal end, which comprised motifs 3 and 6. Within Group III, SiCSP8 and 6 exhibited a degenerate motif 6 and SiCSP2 a much shorter C-terminus as motif 6 appeared shuffled toward the N-terminus, where it replaced motif 4. The similarities and differences observed amongst SiCSPs within the core motifs, the N-terminal and C-terminal ends suggest that SiCSP functional diversification was mainly driven by gradual changes occurring at the periphery of the protein core via point mutations, indels and domain shuffling (Bagowski et al., 2010). Nevertheless, the consequences of divergences and convergences of SiCSP expression, as well as the motif analyses that likely reflects structural and functional properties, will only be understood as more information concerning their ligand binding affinities and downstream interacting partners are uncovered.

Our data provide a foundation for future exploration of the functional roles of insect CSPs and suggest that like OBPs, CSPs participate in diverse biological processes as ligand carriers. The systematic expression profiles of both families of SiCSPs and SiOBPs are now available and can be used to identify targets that may participate in specific physiological processes. In addition, these data can be used as a basis for exploring factors that may contribute to ant colony organization and development, self/non-self-recognition and communication, and/or identify potential targets for ant control, i.e., attractants and/or repellents. Combining these expression data with the ligand binding profiles of these proteins is a much-needed future step in their functional characterization (e.g., behavioral studies combined with RNAi). Such ligands may be derived from the environment during chemosensation or endogenously within the organism. In addition, the differential expression of the *CSPs* and *OBPs* in ant castes may be one defining feature or signature that can account for the different behavioral responsiveness of each caste in terms of reacting to chemical cues that lead to task allocation, mating, and other features of the social nature of this ant species.

## Data Availability Statement

All the data supporting the findings is contained within the article and/or Supplementary Materials.

## Author Contributions

NK, AW, WZ, AO-U, and JB initiated the project, conceived and designed the study. WZ, AW, AO-U, and JB performed the samples collection, library constructions, q-RT-PCR, data processing, bioinformatic analyses, data processing, and contributed to revisions of the manuscript. JB and YX provided resources and/or materials for the study. NK, AO-U, and YX wrote the manuscript, contributed to bioinformatic analyses, data processing, and data interpretation. All authors contributed to the article and approved the submitted version.

## Conflict of Interest

The authors declare that the research was conducted in the absence of any commercial or financial relationships that could be construed as a potential conflict of interest.

## Footnotes

1 https://software.broadinstitute.org/Morpheus

2 http://www.cbs.dtu.dk/services/SignalP/

3 http://gsds.cbi.pku.edu.cn

4 http://meme-suite.org/tools/meme

## Supplementary Material

Supplementary Figure 1 Homogenous subsets yielded by the comparison of means via HSD Tukey’s test within developmental stage. Within each developmental stage, different letters indicate significant differences at *P* < 0.05. *SiCSPs* for each developmental stage are arranged from low to high levels of expression.

Supplementary Figure 2 Motif analyses of CSPs. **(A)** Positions of indicated motifs in the amino acid sequences of 92 CSPs. **(B)** Most common amino acid motifs identified in 92 CSPs using the MEME server (version 4.11.2, http://meme-suite.org/tools/meme). The size (height) of the amino acid represents the degree of conservation of that amino acid in the consensus sequence.

Supplementary Table 1 List of SiCSP genes.

Supplementary Table 2 List of primers.

Supplementary Table 3 Validation of primer pairs.

Supplementary Table 4 Proteins used in phylogenetic analyses.

Supplementary Datasheet 1 Ct values yielded by the RT-qPCR analyses, and absolute and normalised number of transcripts for the developmental stages, workers, males and female alates.
