# Supplementary material for: Red Imported Fire Ant (Solenopsis invicta) Chemosensory Proteins Are Expressed in Tissue, Developmental, and Caste-Specific Patterns
Source: Front Physiol. 2020 Oct 23;11:585883. doi: 10.3389/fphys.2020.585883 (PMC7646262; doi:10.3389/fphys.2020.585883)
Supplement: Supplementary Table 1 — List of SiCSP genes. [file Table_1.DOCX]

**Supplemental Materials**

**Red imported fire ant (*Solenopsis invicta*) chemosensory proteins are expressed in tissue, developmental, and caste-specific patterns**

Arun Wanchoo^2§^, Wei Zhang^1,2§^, Almudena Ortiz-Urquiza^2,3§^*, John Boswell^2^, Yuxian Xia^1^, and Nemat O. Keyhani^1,2^*

^§^These three authors contributed equally to the work.

**Supplemental Tables**

**Table S1. List of *S. invicta* CSP genes**

| Name | AA Length | GenBank accession number | Cysteine | Signal Peptide |
| --- | --- | --- | --- | --- |
| SiCSP1 | 100 | XP_011163783 | 4 | 21 |
| SiCSP2 | 91 | EFZ17972 | 4 | 29 |
| SiCSP3 | 110 | XP_011166108 | 4 | 19 |
| SiCSP4 | 109 | XP_011160225 | 5 | 22 |
| SiCSP6 | 100 | XP_011164980 | 4 | 18 |
| SiCSP7 | 108 | EFZ17227 | 4 | 16 |
| SiCSP8 | 108 | XP_011160350 | 4 | 14 |
| SiCSP9 | 90 | EFZ11660 | 4 | 22 |
| SiCSP10 | 91 | XP_011168346 | 4 | 22 |
| SiCSP11 | 99 | XP_011160349 | 6 | 21 |
| SiCSP12 | 89 | XP_011164970 | 4 | 22 |
| SiCSP13 | 91 | XP_011160273 | 5 | 23 |
| SiCSP14 | 102 | EFZ22773 | 5 | 22 |
| SiCSP15 | 100 | EFZ17504 | 6 | 21 |
| SiCSP16 | 99 | XP_011172450 | 4 | 20 |
| SiCSP17 | 100 | XP_011160224 | 4 | 20 |
| SiCSP18 | 98 | XP_011164990 | 4 | 20 |
| SiCSP19 | 91 | XP_011160348 | 4 | 23 |
| SiCSP20 | 91 | XP_011159610 | 6 | 23 |
| SiCSP21 | 99 | XP_011163782 | 4 | 20 |
| SiCSP22 | 90 | XP_011160348 | 4 | 23 |

**Table S2. List of primers used for quantitative RT-PCR primers**

|  | Forward | Reverse |
| --- | --- | --- |
| SiCSP1 | TGCTGGCCATTCTGGCTGT | CGAAGCTCATCGTTCGGCA |
| SiCSP2 | CAACTGAACATAGCCCTGAG | GATCTGACGTATCTCTTCGG |
| SiCSP3 | CTGCTGAAGCTTTAGAGGC | CATCCTCATACTTCTTGGCG |
| SiCSP4 | GGATGTAGCGAGACGCAGAA | TAACGCCGTCGATATGTCCC |
| SiCSP6 | CCTCCAGAATAATCGCGTTC | GATCGTATTTGGCGACGA |
| SiCSP7 | CGAGAAGGTCATCCGGTATC | GGCCATTGGGATCATACTTC |
| SiCSP8 | GATAAAGGTCCCTGTACTGC | CTCTCGAGAAATTCTGCCC |
| SiCSP9 | CTTGCAGAAGACCTCCATAG | CCAAGTATCGGGTTGGTTC |
| SiCSP10 | AAGAGTACCGTGCGCAAAAG | TCCAAGTATCGGGTTCGTTC |
| SiCSP11 | CGTAACAGCCGATGCAAAGTTC | ACGGCAGCTTGCCATTTATC |
| SiCSP12 | GGACCGTGCACACTAGAACA | AGTGCATCTTCTGCAGTTGG |
| SiCSP13 | TATCGCGTTGCTCGTTGTTG | TTCTGCGCACGGTTCTCTTT |
| SiCSP14 | ATGCGTTCTCGCGAAAGAAGAG | ACGTGCACGGTGTTATTCCC |
| SiCSP15 | TTGCACTAATGTGCGTTGCC | GTTACGCACGACGATGTTCC |
| SiCSP16 | AAAACAGCCGATGCGAAG | AGCTTGCCACTCATCAGGTT |
| SiCSP17 | AGGGGTTTTTGCAGATGCTC | GAGAGCCTGCCATTCATCAG |
| SiCSP18 | CCGCAGATGCGAAATTCT | GAAGGCTTCCCATTGTTCAG |
| SiCSP19 | AAAGGACCGTGCTCAGAAGA | GGCTTCGTTCTGTTCGTACC |
| SiCSP20 | CGTACTGATGTGCGTTCTTGG | CTTCTGTACACGGTGCTCTT |
| SiCSP21 | ATCGCCATGCGTAACAG | CCTCAGATTTTGCAACGGC |
| SiCSP22 | GCGACAGAAAGCAGTCGAT | TCCAAGTATCGGGTTGGTTC |
| EF1-α | AAGAGAACCCGAAAGCCATT | GCCTCAACGCACATAGGTTT |
| GAPDH | AAGCTGTGGCGTGATGGCCG | AGGAGGCAGGCTTGGCGAGT |

**Table S3. Validation of RT-PCR Primer sets**.

| Gene | Amplicon size (bp) | Exon Location | Approximate size on gel (bp)^1^ | Optimal ratio of primers | T_M_ (°C) | Efficiency (%) |
| --- | --- | --- | --- | --- | --- | --- |
|  |  |  |  | [3 µM stock] |  |  |
|  |  |  |  | (µL: µL) |  |  |
| *SiCSP1* | 106 | 1-2 | 100 | 1:1 | 62.2 | 102.21 |
| *SiCSP2* | 165 | 1-2 | 200 | 1:1 | 55.8 | 101.14 |
| *SiCSP3* | 192 | 1-2 | 200 | 1:1 | 56 | 102.7 |
| *SiCSP4* | 125 | 1-2 | 100 | 1:1 | 59.7 | 84.91 |
| *SiCSP6* | 221 | 1-2 | 200 | 1:2 | 55 | 95.77 |
| *SiCSP7* | 80 | 1-2 | 100 | 1:1 | 59.2 | 93.8 |
| *SiCSP8* | 167 | 1-2 | 200 | 1:1 | 55.2 | 112.86 |
| *SiCSP9* | 246 | 1-2 | 200 | 2:2 | 55.3 | 113.81 |
| *SiCSP10* | 149 | 1-2 | 100 | 1:1 | 59.9 | 96.14 |
| *SiCSP11* | 150 | 1-2 | 200 | 1:2 | 59.2 | 108.44 |
| *SiCSP12* | 78 | 1-2 | 100 | 1:2 | 59 | 90.6 |
| *SiCSP13* | 169 | 1-2 | 200 | 1:1 | 59.6 | 111.45 |
| *SiCSP14* | 128 | 1-2 | 100 | 1:2 | 61 | 91.96 |
| *SiCSP15* | 139 | 1-2 | 100 | 1:1 | 59.6 | 104.12 |
| *SiCSP16* | 144 | 1-2 | 100 | 1:1 | 59.9 | 91.01 |
| *SiCSP17* | 121 | 1-2 | 100 | 1:2 | 60.9 | 97.43 |
| *SiCSP18* | 143 | 1-2 | 100 | 1:1 | 59.7 | 98.52 |
| *SiCSP19* | 138 | 1-2 | 100 | 1:1 | 59.7 | 93.84 |
| *SiCSP20* | 143 | 1-2 | 100 | 2:1 | 57.6 | 104.21 |
| *SiCSP21* | 173 | 1-2 | 200 | 1:1 | 55 | 112.28 |
| *SiCSP22* | 179 | 1-2 | 200 | 1:2 | 59.1 | 100.04 |
| *EF1-α* | 79 | 1-2 | 100 | 1.5:1.5 | 60 | 108.24 |
| *GAPDH* | 184 | 1-2 | 200 | 2:2 | 60 | 100.35 |

^1^Determined by agarose gel electrophoresis.

**Table S4. List of proteins used in phylogenetic analyses and nomenclature correspondence for Fig. 1.**

|  | CHEMOSENSORY PROTEIN (CSP) | | | |  |
| --- | --- | --- | --- | --- | --- |
| SPECIES | Keyhani^1^ | Kulmuni et al. [1] | McKenzie et al [2] | Hojo et al [3] | BLAST ACCESION NUMBER |
| *Acromyrmex echinatior* | AeCSP1 | AeCSP7 |  | AeCSP1 | EGI69116 |
|  | AeCSP2 | AeCSP5 |  | AeCSP2 | EGI70267 |
|  | AeCSP3 | AeCSP3 |  | AeCSP3 | EGI70269 |
|  | AeCSP4 | AeCSP1 |  | AeCSP4 | XP_011053888 |
|  | AeCSP6 | AeCSP4 |  | AeCSP6 | XP_011068172 |
|  | AeCSP7 | AeCSP2 |  | AeCSP7 | XP_011050119 |
|  | AeCSP8 | AeCSP6 |  | AeCSP8 | EGI60804 |
|  | AeCSP9 | AeCSP9 |  | AeCSP9 | XP_011068665 |
|  | AeCSP10 | AeCSP10 |  | AeCSP10 | XP_011068664 |
|  | AeCSP11 | AeCSP11 |  | AeCSP11 | XP_011053869 |
|  | AeCSP12 | AeCSP12 |  | AeCSP12 | EGI64540 |
|  | AeCSP13 | AeCSP13 |  | AeCSP15 | EGI66215 |
|  | AeCSP14 | AeCSP14 |  | AeCSP14 | EGI64541 |
|  | AeCSP21 | AeCSP8 |  | AeCSP21 | EGI64542 |
| *Camponotus floridanus* | CfCSP1 | CfCSP7 | CfCSP1 |  | XP_011265982 |
|  | CfCSP2 | CfCSP5 | CfCSP2 |  | XP_011269585 |
|  | CfCSP3 | CfCSP3 | CfCSP3 |  | XP_011255580 |
|  | CfCSP4 | CfCSP1 | CfCSP4 |  | XP_011255579 |
|  | CfCSP5 | CfCSP13 | CfCSP5 |  | XP_011258405 |
|  | CfCSP6 | CfCSP4 | CfCSP6 |  | XP_011258406 |
|  | CfCSP7 | CfCSP2 | CfCSP7 |  | EFN65878 |
|  | CfCSP8 | CfCSP6 | CfCSP8 |  | XP_011261803 |
|  | CfCSP9 | CfCSP9 | CfCSP9 |  | XP_011261802 |
|  | CfCSP10 | CfCSP10 | CfCSP10 |  | XP_011267897 |
|  | CfCSP11 | CfCSP11 | CfCSP11 |  | XP_011250433 |
|  | CfCSP12 | CfCSP12 | CfCSP12 |  | XP_011269548 |
|  | CfCSP13 | CfCSP8 | CfCSP13 |  | XP_011253063 |
| *Camponotus japonicus* | CjCSP1 |  |  | CjCSP1 | BAS29775 |
|  | CjCSP2 |  |  | CjCSP2 | BAS29776 |
|  | CjCSP3 |  |  | CjCSP3 | BAS29777 |
|  | CjCSP4 |  |  | CjCSP4 | BAS29778 |
|  | CjCSP5 |  |  | CjCSP5 |  |
|  | CjCSP6 |  |  | CjCSP6 |  |
|  | CjCSP7 |  |  | CjCSP7 | BAS29779 |
|  | CjCSP8 |  |  | CjCSP8 |  |
|  | CjCSP9 |  |  | CjCSP9 |  |
|  | CjCSP10 |  |  | CjCSP10 |  |
|  | CjCSP12 |  |  | CjCSP12 | BAS29780 |
|  | CjCSP13 |  |  | CjCSP13 | BAS29781 |
| *Harpegnathos saltator* | HsCSP1 | HsCSP3 | HsCSP1 |  | XP_011151735 |
|  | HsCSP2 | HsCSP8 | HsCSP2 |  | XP_011154714 |
|  | HsCSP3 | HsCSP1 | HsCSP3 |  | XP_011153546 |
|  | HsCSP4 | HsCSP5 | HsCSP4 |  | XP_011153548 |
|  | HsCSP5 | HsCSP6 | HsCSP5 |  | XP_011152650 |
|  | HsCSP6 | HsCSP11 | HsCSP6 |  | XP_011152649 |
|  | HsCSP7 | HsCSP9 | HsCSP7 |  | EFN85227 |
|  | HsCSP8 | HsCSP10 | HsCSP8 |  | XP_011138157 |
|  | HsCSP9 | HsCSP7 | HsCSP9 |  | EFN81741 |
|  | HsCSP10 | HsCSP2 | HsCSP10 |  | XP_011137361 |
|  | HsCSP11 | HsCSP12 | HsCSP11 |  | XP_011137370 |
|  | HsCSP14 | HsCSP4 | HsCSP14 |  | XP_011143827 |
| *Solenopsis invicta* | SiCSP1 | SiCSP7 | SiCSP1 |  | XP_011160273 |
|  | SiCSP2 | SiCSP5 | SiCSP2 |  | EFZ11660 |
|  | SiCSP3 | SiCSP3 | SiCSP3 |  | XP_011163782 |
|  | SiCSP4 | SiCSP1 | SiCSP4 |  | XP_011163783 |
|  | SiCSP6 | SiCSP4 | SiCSP6 |  | XP_011166108 |
|  | SiCSP7 | SiCSP2 | SiCSP7 |  | XP_011172450 |
|  | SiCSP8 | SiCSP6 | SiCSP8 |  | EFZ17227 |
|  | SiCSP9 | SiCSP9 | SiCSP9 |  | XP_011164980 |
|  | SiCSP10 | SiCSP10 | SiCSP10 |  | XP_011160350 |
|  | SiCSP11 | SiCSP11 | SiCSP11 |  | XP_011160226 |
|  | SiCSP12 | SiCSP12 | SiCSP12 |  | XP_011164990 |
|  | SiCSP13 | SiCSP13 | SiCSP13 |  | EFZ22773 |
|  | SiCSP14 | SiCSP14 | SiCSP14 |  | XP_011159610 |
|  | SiCSP15 | SiCSP15 | SiCSP15 |  | XP_011160225 |
|  | SiCSP16 | SiCSP16 | SiCSP16 |  | EFZ17972 |
|  | SiCSP17 | SiCSP17 | SiCSP17 |  | EFZ17504 |
|  | SiCSP18 | SiCSP18 | SiCSP18 |  | XP_011168346 |
|  | SiCSP19 | SiCSP19 | SiCSP19 |  | XP_011160348 |
|  | SiCSP20 | SiCSP20 | SiCSP20 |  | XP_011160349 |
|  | SiCSP21 | SiCSP21 | SiCSP21 |  | XP_011160224 |
|  | SiCSP22 | SiCSP8 | SiCSP24 |  | XP_011164970 |
| *Drosophila melanogaster* | DmCSP1 |  |  |  | AAN71635 |
|  | DmCSP2 |  |  |  | NP_611990 |
|  | DmCSP3 |  |  |  | NP_524966 |
|  | DmCSP4 |  |  |  | NP_524121 |
| *Drosophila grimshawi* | DgCSP1 |  |  |  | XP_001987083 |
|  | DgCSP2 |  |  |  | XP_001987660 |
|  | DgCSP4 |  |  |  | XP_001991170 |
|  | DgCSP3 |  |  |  | XP_001987061 |
| *Apis mellifera* | Amel1 |  | Amel1 | Amel1 | NP_001071288 |
|  | Amel2 |  | Amel2 | Amel2 | NP_001071278 |
|  | Amel3 |  | Amel3 | Amel3 | NP_001011583 |
|  | Amel4 |  | Amel4 | Amel4 | NP_001071282 |
|  | Amel5 |  | Amel5 | Amel5 | NP_001072129 |
|  | Amel6 |  | Amel6 | Amel6 | NP_001071287 |
| *Polistes canadensis* | Pcan1 |  | Pcan1 |  | XP_014604789 |
|  | Pcan2 |  | Pcan2 |  | XP_014605111 |
|  | Pcan3 |  | Pcan3 |  | XP_014608488 |
|  | Pcan4 |  | Pcan4 |  | XP_014608404 |
|  | Pcan5 |  | Pcan5 |  | XP_014608124 |
|  | Pcan7 |  | Pcan7 |  | XP_014607673 |
|  | Pcan9 |  | Pcan9 |  | XP_014604789 |

^1^ This paper

1. Kulmuni J, Wurm Y, Pamilo P: **Comparative genomics of chemosensory protein genes reveals rapid evolution and positive selection in ant-specific duplicates**. *Heredity* 2013, **110**(6):538-547.

2. McKenzie SK, Oxley PR, Kronauer DJC: **Comparative genomics and transcriptomics in ants provide new insights into the evolution and function of odorant binding and chemosensory proteins**. *BMC Genomics* 2014, **15**.

3. Hojo MK, Ishii K, Sakura M, Yamaguchi K, Shigenobu S, Ozaki M: **Antennal RNA-sequencing analysis reveals evolutionary aspects of chemosensory proteins in the carpenter ant, *Camponotus japonicus***. *Sci Rep-Uk* 2015, **5**.
